# Unexpected Digit: Isolated Acrometastasis to the Fifth Finger in Advanced Lung Cancer

**DOI:** 10.7759/cureus.85646

**Published:** 2025-06-09

**Authors:** Zakaria Alameddine, Parima Saxena, Micha Gooden, Farish Mohamed, Elvira Neculiseanu

**Affiliations:** 1 Oncology, State University of New York Downstate Health Sciences University, Brooklyn, USA; 2 Oncology, State University of New York Downstate Medical Center, Brooklyn, USA

**Keywords:** acrometastases, amputation, bone metastases, fifth digit, lung squamous cell carcinoma

## Abstract

Acrometastasis is a rare manifestation of metastatic disease that affects the distal extremities and can occur as a complication of non-small-cell lung cancer. It is often associated with advanced disease and may coincide with other skeletal or systemic metastases. Management options typically include a combination of surgical intervention, radiation therapy, and systemic treatment. This report presents a case of an isolated acrometastatic lesion in the fifth digit of a patient with stage IV squamous cell carcinoma, without evidence of additional metastatic bone involvement. We explore diagnostic and therapeutic strategies for such presentations and emphasize the importance of a multidisciplinary approach in optimizing patient care. We also address the underlying pathophysiology and potential mechanisms contributing to acrometastatic spread.

## Introduction

Acrometastases are secondary bone lesions that develop distally to the elbow or knee in the upper and lower extremities, respectively [1]. They are considered to be rare entities as they account for 0.1% of all metastatic bone lesions, with 10% serving as the initial sign of malignancy [2]. The third digit and thumb are the most affected among the fingers, whereas the fifth is the least frequently involved [3,4]. Lung cancer represents the most prevalent primary site, accounting for 30-40% of cases [5]. This is followed closely by renal cell cancer, breast cancer, and colon cancer [2]. Due to its rarity and nonspecific presentation, it poses a diagnostic challenge, as it can mimic multiple benign conditions. Diagnostic workup typically commences with an X-ray and is eventually confirmed through histologic examination of biopsy specimens [2,5]. Here, we report a rare case of squamous cell lung cancer initially staged as IIIB, treated with chemoradiation, that rapidly progressed to widespread metastatic disease with an unusual acrometastasis to the right fifth finger as the sole site of osseous involvement.

## Case presentation

A 76-year-old man with stage IV non-small-cell lung cancer (NSCLC) presented to the medical oncology clinic for a routine follow-up, complaining of multiple symptoms that included cough, shortness of breath, dizziness, and blurry vision associated with pain and swelling of his right fifth finger. Four months prior, he was diagnosed with stage IIIB squamous cell lung cancer without targetable mutation on next-generation sequencing (NGS) and a PDL-1 score of 10%. He received seven weeks of concurrent chemoradiation with carboplatin and paclitaxel, followed by a post-treatment positron emission tomography/computed tomography (PET/CT) to assess the patient's response to therapy, which revealed newly developed left hilar and right superior paratracheal lymphadenopathies, along with newly developed hypermetabolic bilateral lobar liver lesions. His past medical history includes hypertension, hyperlipidemia, and stage 3A chronic kidney disease. His past surgical history includes an infrarenal abdominal aortic aneurysm for which he underwent an endovascular repair. He is a heavy smoker but stopped consumption after his lung cancer diagnosis, and he denies allergies to drugs or food. His physical exam was remarkable for swelling, erythema, and bleeding from his right fifth distal phalanx associated with a violaceous discoloration.

**Figure 1:**
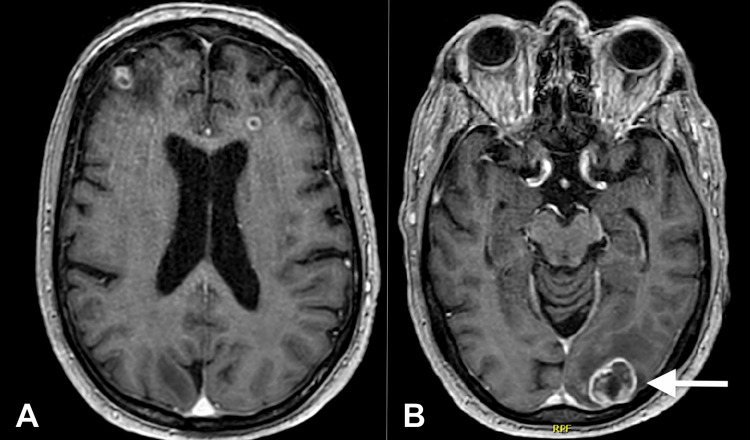
Brain MRI was performed with and without gadolinium contrast. Images displayed (A) and (B) represent post-contrast sequences demonstrating multiple supratentorial heterogeneously enhancing and ring-enhancing cortical-based lesions, some demonstrating internal blood products. Findings are highly concerning for hemorrhagic intracranial metastases. The largest metastasis is within the left occipital lobe, measuring 2.5 × 2.1 × 2.1 cm (B, white arrow). Vasogenic edema is seen surrounding the intracranial metastases and is most pronounced within the left occipital lobe. RPF: Reference position frame

He was sent to the emergency department for his aforementioned symptoms. His laboratory tests, including a complete blood count and a comprehensive metabolic panel, were within normal ranges. A magnetic resonance imaging (MRI) of his brain showed multiple supratentorial heterogeneously enhancing and ring-enhancing cortical-based lesions, highly concerning for hemorrhagic intracranial metastases. The largest of which is located within the left occipital lobe, measuring 2.5 cm × 2.1 cm × 2.1 cm, accompanied by vasogenic edema surrounding the intracranial metastases (Figure 1).

Considering the multiplicity of his lesions, age, comorbidities, and frailty, the neurosurgery team deemed that the resection of even the largest occipital lesion would be poorly tolerated in this patient. Radiation oncology was consulted, and the patient was started on dexamethasone while being planned for whole brain radiation therapy.

**Figure 2:**
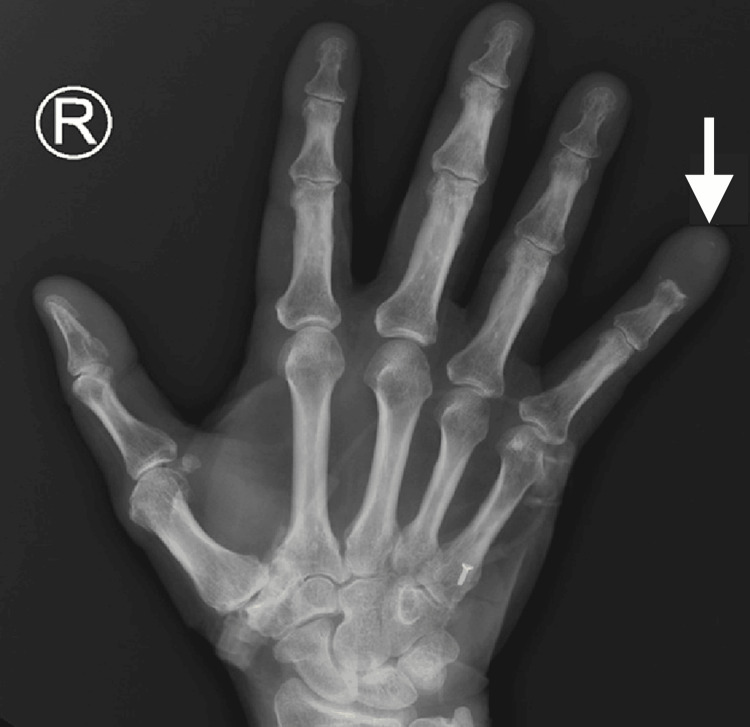
Diagnostic anteroposterior (AP) X-ray imaging of the right hand. Destructive lesion of the fifth distal phalanx (arrow).

**Figure 3:**
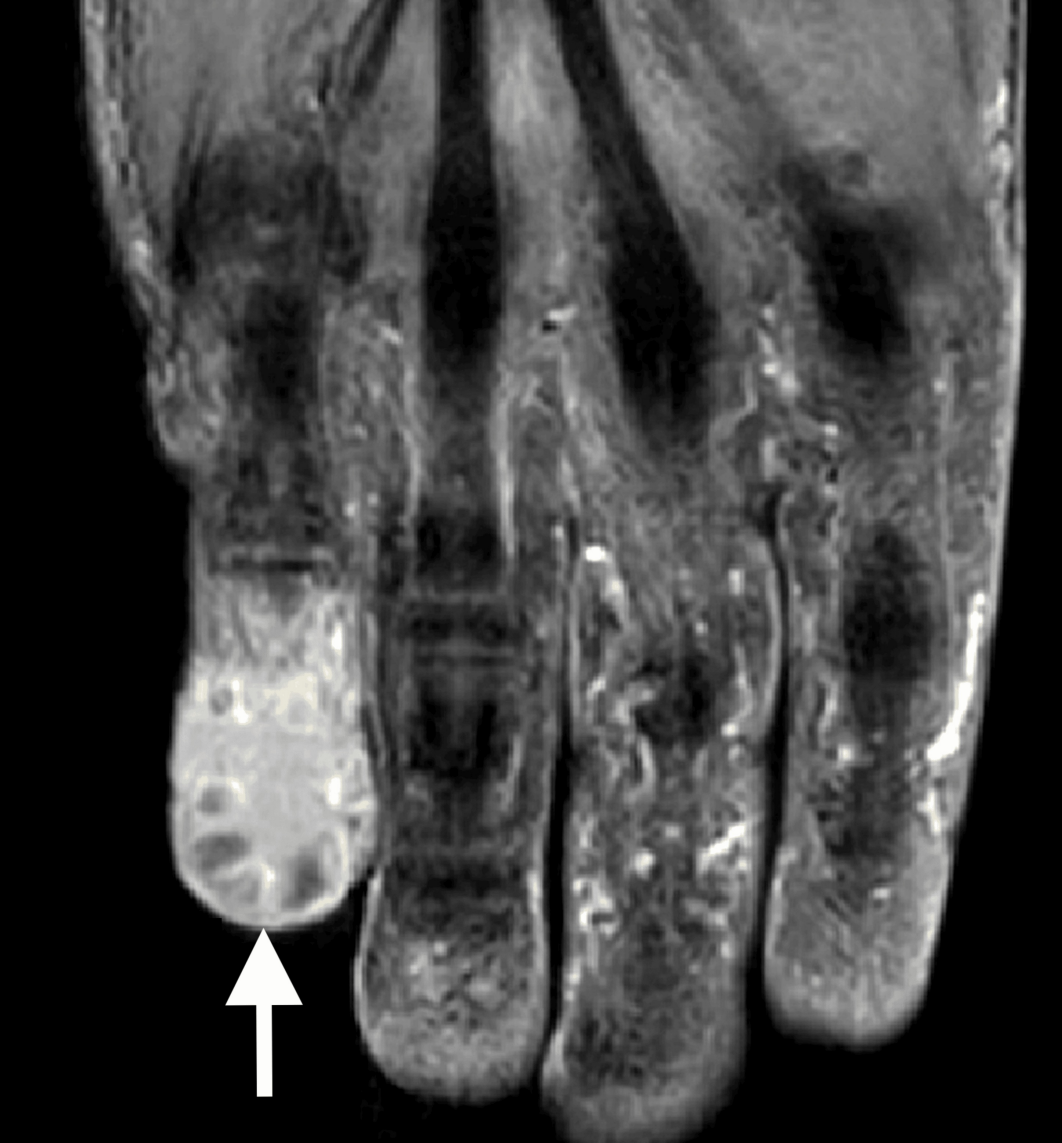
MRI of the right hand with and without gadolinium. Imaging demonstrates an expansile lytic lesion in the distal phalanx of the small finger with involvement of the middle phalanx (arrow).

**Figure 4:**
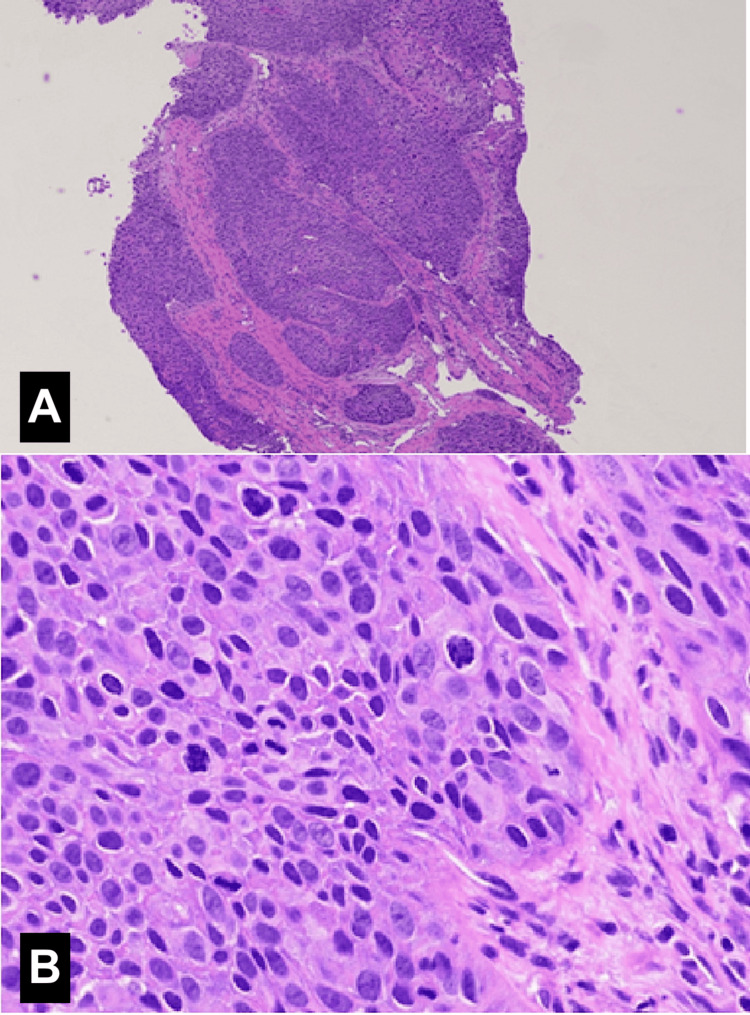
Histopathology images stained with hematoxylin and eosin (H&E) of an open biopsy of the right fifth finger. (A) At 4× magnification, infiltrative nests and islands of malignant cells with an invasive growth pattern are observed; (B) At 40× magnification, intercellular bridges, eosinophilic cytoplasm, and prominent nucleoli, along with nuclear pleomorphism, confirm the presence of squamous cell carcinoma.

**Figure 5:**
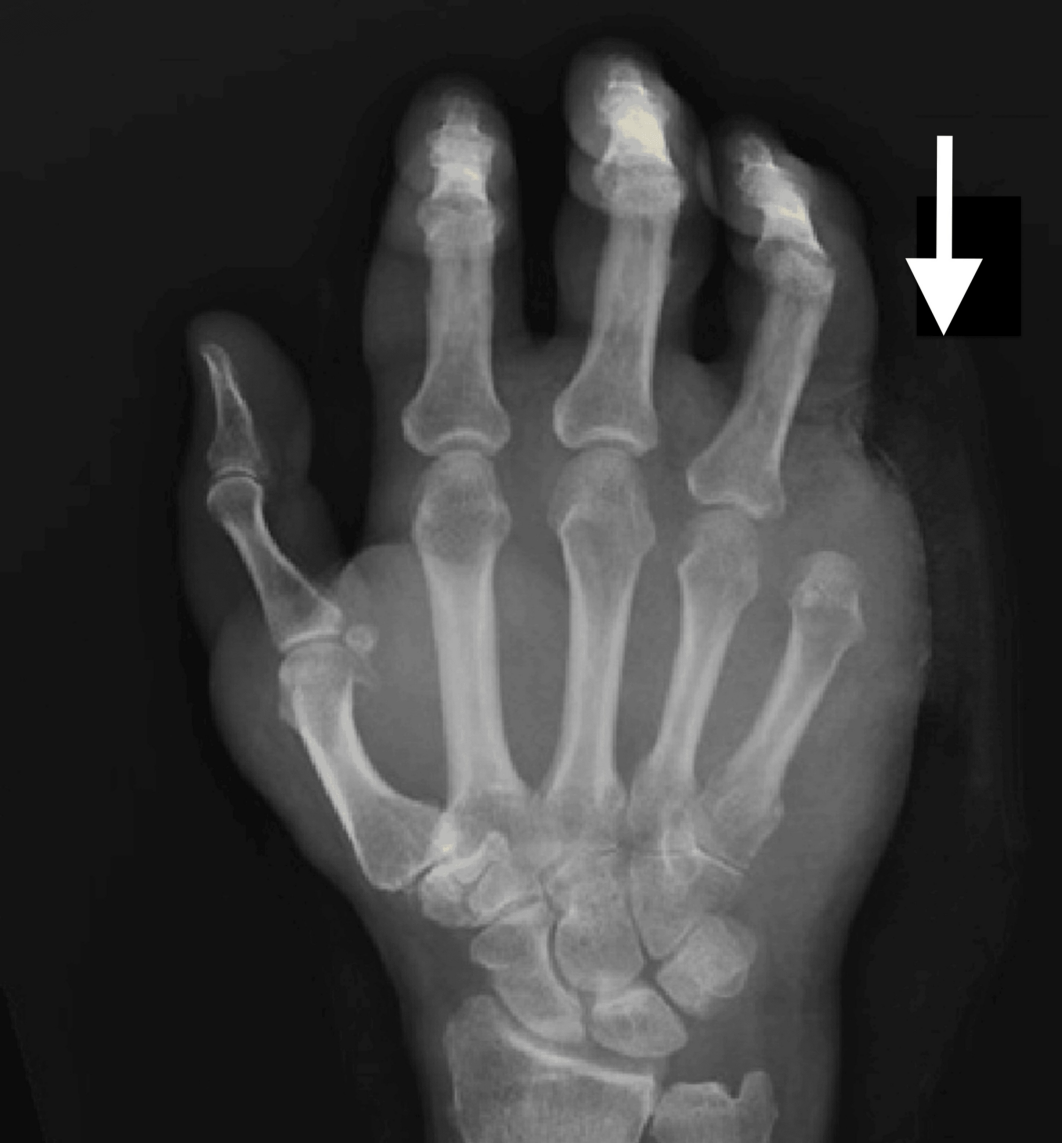
Postoperative X-ray imaging of the right hand following the amputation of the right fifth finger with disarticulation through the metacarpophalangeal joint. The arrow denotes the digit that was amputated.

Meanwhile, an X-ray of the right hand obtained to evaluate the swelling and pain of his right fifth finger revealed a destructive lesion of the fifth distal phalanx (Figure 2). MRI for further characterization revealed the presence of an expansile lytic lesion of the distal phalanx with involvement of the middle phalanx (Figure 3). Additionally, a bone survey did not demonstrate additional bone lesions. After a fine needle aspirate (FNA) failed to provide a definitive cytologic diagnosis, an open biopsy of the right fifth finger was performed by the orthopedics team, which confirmed the presence of invasive squamous cell carcinoma (Figure 4). His condition was managed by a palliative amputation with disarticulation through the right fifth metacarpophalangeal joint (Figure 5).

The patient was eventually started on systemic treatment for his metastatic disease after his whole brain radiation therapy was completed with ipilimumab and nivolumab, but unfortunately, he died shortly after due to complications from a combination of cancer progression and treatment-related side effects.

## Discussion

Metastasis distal to the elbow or knee, defined as acrometastasis, is a rare occurrence, accounting for approximately 0.1% of overall metastases [2]. The lung is frequently implicated in this type of metastasis, likely due to its direct access to the systemic circulation. This supports the theory that tumor cells primarily spread to distal extremities through hematogenous propagation rather than the lymphatic system [2]. The predominant histological subtype is squamous cell carcinoma [6]. Additionally, acrometastases from the lung predominantly affect the hands, and this pattern is attributed to the diaphragm acting as a boundary, where supradiaphragmatic tumors, such as lung cancer, can directly access the systemic arterial circulation, thereby facilitating spread to the hands. In contrast, infradiaphragmatic tumors, such as renal and colorectal cancers, tend to metastasize to the feet, possibly via retrograde venous pathways [2].

The dominant hand is usually affected, and this is hypothesized to be due to a combination of increased circulation, repetitive trauma, and chemotactic factors [5,7]. The distal phalanx is most involved, possibly because tumor emboli are more likely to lodge in areas with slower blood flow [2,4]. Unlike breast cancer, acrometastatic lesions to the hand from a lung primary tend to involve a single bone [3]. This aligns with the presentation in our patient, who developed a single bone acrometastasis from squamous cell lung cancer involving the distal phalanx of the dominant right hand. Interestingly, our patient's metastasis involved the fifth finger, which is considered the least commonly affected digit. However, in the hand overall, the carpus is generally regarded as the rarest site of metastasis [2,3].

A retrospective study by Hosaka et al. evaluating patients with bone metastases found that approximately 21% of patients with disseminated disease had a single bone lesion, suggesting that bone involvement in metastatic cancer is often accompanied by additional skeletal lesions [8]. This contrasts with our case, where the patient had a solitary bone lesion.

Clinical manifestation includes pain, erythema, and swelling, but it can be misleading as it often mimics benign conditions such as infectious, rheumatological, or orthopedic disorders [9]. A systematic review by Dow et al. reported that nearly half of the reported wrist and hand lesions were initially misdiagnosed, most frequently as infections [4].

Workup usually begins with a plain radiograph, which characterizes the lesions as lytic, blastic, or mixed based on their radiographic appearance. Patients with lung cancer, such as our patient, typically present with a lytic lesion [3]. MRI is the gold standard, but histologic confirmation requires a biopsy [2]. Although bone lesions in patients with known cancer are often presumed metastatic, especially if imaging is typical and with multiple other sites, a biopsy is advised if features are atypical or the location is unusual, such as in acrometastases [10].

There is no standard protocol for management [9], but amputation, whether digital or ray, seems to be the most employed [11], especially in cases involving the distal phalanx [2]. Radiation therapy can also be used mostly as a palliative measure to alleviate pain and preserve the function of the limb. It is also utilized in certain circumstances where surgery is not feasible, such as in cases of proximal tumors [11,12]. Some experts advise against radiation in cases of complete destruction of the bone [13]. Systemic therapy is primarily employed to manage overall disease burden in patients with acrometastases, rather than as a sole measure. ​In our case, after a thorough discussion with the patient, he expressed a strong desire to prevent further disease progression in his finger and to palliate pain. Given the distal location of the lesion and the extensive bone loss, we elected for palliative amputation.​

Prognosis is typically poor in such patients. The median survival is approximately six months [9], with comparable survival outcomes between patients treated with radiotherapy alone and those who underwent amputation for lesions in similar anatomical locations [4]. Our patient succumbed within a month of his procedure, secondary to complications from his progressive disease and treatment-related side effects.

## Conclusions

Acrometastasis of the hand is exceedingly rare and continues to pose a diagnostic challenge for many physicians. Thus, a high index of suspicion is crucial for diagnosis since these lesions may represent the only site of bone involvement, even amid widespread metastatic disease. Our case highlights the importance of early recognition and the need for a multidisciplinary approach to achieve optimal management.

## References

[1] Pușcașu AI, Moinard-Butot F, Antoni D, Schott R, Somme L (2023). Solitary acrometastasis of the phalanx as initial presentation of an oligometastatic Kirsten rat sarcoma viral oncogene homolog-mutated lung adenocarcinoma: a case report. J Med Case Rep.

[2] Stomeo D, Tulli A, Ziranu A, Perisano C, De Santis V, Maccauro G (2015). Acrometastasis: a literature review. Eur Rev Med Pharmacol Sci.

[3] Flynn CJ, Danjoux C, Wong J (2008). Two cases of acrometastasis to the hands and review of the literature. Curr Oncol.

[4] Dow T, Davis C, ElAbd R, Lalonde D, Williams J (2024). Cancer metastases to the hand: a systematic review and meta-analysis. Hand (N Y).

[5] Jaber SK, Hashem GN, Mouawad JA, Kalaji JG, Akl JK (2024). Index finger acrometastasis: a unique lung cancer case report. Int J Surg Case Rep.

[6] Sahoo TK, Das SK, Majumdar SK, Senapati SN, Parida DK (2016). Digital acrometastasis as initial presentation in carcinoma of lung a case report and review of literature. J Clin Diagn Res.

[7] Kumar N, Kumar R, Bera A (2011). Palliative and supportive care in acrometastasis to the hand: case series. Indian J Palliat Care.

[8] Hosaka S, Katagiri H, Honda Y, Wasa J, Murata H, Takahashi M (2016). Clinical outcome for patients of solitary bone only metastasis. J Orthop Sci.

[9] Hossain S, Baralo B, Thota V, Nijhum T, Mulla S, Thar YU (2021). A rare case of phalanx acrometastasis of lung adenocarcinoma. Chest.

[10] Raphael B, Hwang S, Lefkowitz RA, Landa J, Sohn M, Panicek DM (2013). Biopsy of suspicious bone lesions in patients with a single known malignancy: prevalence of a second malignancy. AJR Am J Roentgenol.

[11] Ferini G, Zagardo V, Viola A (2023). Considerations on surgery invasiveness and response and toxicity patterns in classic palliative radiotherapy for acrometastases of the hand: a hint for a potential role of stereotactic body radiation therapy? A case report and literature review. Front Oncol.

[12] Mazur TR, Gach HM, Schiff JP, Ochoa LL, Naughton MJ, Zoberi I (2024). Stereotactic body radiation therapy for palliative reirradiation of acrometastasis in the hand from breast cancer. Adv Radiat Oncol.

[13] Campa T, Fagnoni E, Ripamonti C (2004). Palliative surgery of acrometastases from lung cancer: a case report. Support Care Cancer.

